# Impact of simulated flight conditions on supraventricular and ventricular ectopy

**DOI:** 10.1038/s41598-022-27113-x

**Published:** 2023-01-10

**Authors:** Mark J. Meyer, Irina Mordukhovich, Brent A. Coull, John McCracken, Gregory A. Wellenius, Murray A. Mittleman, Eileen McNeely

**Affiliations:** 1 Department of Mathematics and Statistics, Georgetown University, Washington, DC 20057 USA; 2 Department of Environmental Health, Harvard T. H. Chan School of Public Health, Boston, MA 02115 USA; 3 Department of Biostatistics, Harvard T. H. Chan School of Public Health, Boston, MA 02115 USA; 4 Global Health Institute, Epidemiology and Biostatistics, University of Georgia, Athens, GA 30602 USA; 5 Department of Environmental Health, Boston University School of Public Health, Boston, MA 02118 USA; 6 Department of Epidemiology, Harvard T.H. Chan School of Public Health, Boston, MA 02115 USA

**Keywords:** Epidemiology, Occupational health, Public health

## Abstract

Though billions of passengers and crew travel by air each year and are exposed to altitude equivalents of 7000–8000 feet, the health impact of cabin oxygenation levels has not been well studied. The hypoxic environment may produce ectopic heartbeats that may increase the risk of acute in-flight cardiac events. We enrolled forty older and at-risk participants under a block-randomized crossover design in a hypobaric chamber study to examine associations between flight oxygenation and both ventricular (VE) and supraventricular ectopy (SVE). We monitored participant VE and SVE every 5 min under both flight and control conditions to investigate the presence and rate of VE and SVE. While the presence of VE did not differ according to condition, the presence of SVE was higher during flight conditions (e.g. OR ratio = 1.77, 95% CI: 1.21, 2.59 for SVE couplets). Rates of VE and SVE were higher during flight conditions (e.g. RR ratio = 1.25, 95% CI: 1.03, 1.52 for VE couplets, RR ratio = 1.76, 95% CI: 1.39, 2.22 for SVE couplets). The observed higher presence and rate of ectopy tended to increase with duration of the flight condition. Further study of susceptible passengers and crew may elucidate the specific associations between intermittent or sustained ectopic heartbeats and hypoxic pathways.

## Introduction

Although more than four billion passengers and crew travel by air each year^1^, the health effects of flight are not well studied. Flight is safe and medical events in-flight occur very infrequently, at a rate of approximately 24–130 in-flight medical emergencies per million passengers; cardiac symptoms and events are estimated to cause approximately 7% of in-flight medical events^2^. However, air travel presents a hypoxic environment because of aircraft pressurization to an equivalent of 7000 to 8000 feet altitude^3^. Hence, some passengers or crew may experience health complications, including cardiac arrhythmias, caused by hypoxia during flight^4^. Syncope, which can be caused by cardiac arrhythmias^5^ and hypoxia, is the most frequently occurring medical event in-flight, with syncope or near-syncope comprising an estimated 33% of in-flight medical emergencies^2^.

Excessive supraventricular ectopic activity is associated with increased risk for atrial fibrillation and stroke^6^ while the presence of ventricular ectopy may, in some cases, suggest susceptibility toward life-threatening arrhythmias^7^. However, we are unaware of any previous studies examining the incidence of ectopic heartbeats during or soon after flight or the association between ectopic heartbeats and flight conditions. A few studies report associations, in some cases minimal, between flight exposure, heart rate, and heart rate variability among younger passengers^8^ and crew^9^, groups with generally optimal cardiovascular health. Others report similar associations in older, more susceptible populations^10^. However, people over the age 50 and those with cardiovascular conditions are highly prevalent in the population may also be at heightened risk of flight-induced arrhythmias^11,12^.

To better understand potential associations, we examined the relationship between simulated hypoxic flight conditions and ectopy via a hypobaric chamber study. We hypothesized that we would observe associations between flight conditions and ventricular ectopy (VE) or supraventricular ectopy (SVE).

## Methods

### Study population

We conducted a block-randomized crossover study using a hypobaric chamber to compare the occurrence of ectopy during simulated flight conditions to control. Study inclusion criteria were age over 50 years and not presenting any active health symptoms. Participants were selected to reflect the population of U.S. passengers over age 50, and hence included people with stable cardiac conditions (moderate coronary artery disease or congestive heart failure) classified according to New York Heart Association criteria (n = 13), smokers without cardiac conditions (n = 14), and non-smokers without cardiac conditions (n = 14).

Participants were recruited through medical offices, newspapers, and community centers in Oklahoma City, proximal to the hypobaric chamber at the Civil Aerospace Medical Institute (CAMI). Participants were enrolled after a phone screen by a nurse practitioner determined eligibility based on age and medical profile, and after attending a medical examination documenting oxygen saturation, pulmonary function, electrocardiogram readings, blood pressure, blood tests, height and weight, and health profile.

### Study design

We monitored groups of participants for 2 days in a hypobaric chamber, with a day of rest in between (December 2007 to June 2008). We monitored participants before, during, and immediately after a 4 to 5-h flight simulation, once with flight conditions equivalent to 7000 feet altitude and once during control conditions^13^. Participants were not informed of the exposure condition and exposure order was randomized by group: 30/41 participants received the flight condition on their first day in the chamber. One participant receiving the control condition on the first day could not continue the study because of a reported work conflict. Participants were instructed to behave as they would aboard a flight, and could sleep, read, watch movies, walk about, or talk freely. A research assistant served meals and snacks. Phlebotomists obtained blood specimens for genomic studies. All participants provided informed written consent in accordance with protocols approved by the Human Subjects Committees at the Harvard School of Public Health (Institutional Review Board Protocol #P15170-101), CAMI, and the University of Oklahoma Medical Center. All research was performed in accordance with the Declaration of Helsinki.

### Instrumentation

The chamber resembled a commercial airplane with 12 seats accommodating participants, a medical monitor, and a research assistant. Chamber gauges recorded humidity, temperature, noise, pressure, and carbon dioxide. Participants wore a LifeShirt^TM^ (Vivometrics, Inc., Ventura, CA, United States), a vest made of Lycra material containing sensors (including a single-channel electrocardiograph), to monitor respiratory and cardiac rhythms and waveforms, heart rate, heart rate variability, and respiratory and blood oxygen indices. We previously examined associations between flight condition and heart rate and heart rate variability within this population, showing evidence of time-varying associations between hypoxia, higher heart rate, and lower heart rate variability^10^. An Airliner Cabin Environment Research report demonstrated substantial oxygen desaturation among these participants during flight conditions^13^.

Data were recorded along with respiration measures, blood pressure, and pulse oximetry from peripheral devices and transmitted wirelessly for monitoring cardiac waveforms in real time. Electrocardiogram (ECG) recordings were read offline by trained technicians unaware of exposures and reviewed by cardiologists to identify supraventricular and ventricular ectopy and ECG artefacts^14^.

### Statistical analysis

Observations represent ventricular and supraventricular ectopic beats during five-minute intervals, with ectopy defined as any occurrence of couplets or runs. We inspected data for missing observations, removing six participants who were missing an entire day of observations (five with cardiac conditions and a non-smoker who did not present with a cardiac condition). Measuring ventricular and supraventricular couplets and runs resulted in one observation per measure of ectopy for every five-minute duration. Participants thus had up to 88 measurements available per day for each measure of ectopy. Measurements were recorded before the start of the flight or control condition (pre-condition) and after conditions commenced (post-condition). We examined the presence and rate of ectopy using difference-in-difference regression to compare the change in the odds of at least one event and the change in the rate of events from pre-condition to post-condition between flight and control conditions. The resulting estimated effects represent the ratios of odds ratios (ORs) and rate ratios (RRs), depending on the outcome. The data were correlated because we repeatedly measured ectopy among participants both within exposure day and between; hence, we implemented longitudinal regression techniques to control for within-participant variability. By design, the participants serve as their own controls to account for unmeasured within-participant effects that are stable over the time frame of the study.

To examine the effect of exposure on the presence of ectopy we used a Binomial mixed effects model. From these models, we estimated the OR of ectopy during post-experimental condition versus pre- for both the flight and control days. We estimated the effect of exposure using the ratio of the ORs for each day in our primary analysis. For models evaluating the frequency of ectopic beats, we inspected ectopic beats data for extra-Poisson variability, finding evidence of overdispersion in most outcomes. To account for this, we employed a Negative Binomial mixed effects model, which accommodates the overdispersion in the counts relative to that explained by a Poisson distribution^15^. Thus, for the frequency or count model, we estimated the RR of ectopy during post-experimental condition versus pre- for both the flight and control days. For our primary analysis, we examined the effect of exposure using the ratio of the RRs from both days.

We controlled for repeated sampling by exposure day and accounted for person-level variability. We also controlled for the time of day the participants began monitoring and for the block randomized order of the treatment assignment, hence adjusting for the effect of diurnal variation and for participants growing accustomed to the chamber. We adjusted for blood oxygen at baseline, use of beta blockers, and the presence of a pacemaker. For participant *i*s *j*th 5-min observation of either the presence or count of ectopic beats on the *k*th day in the chamber ($y_{ijk}$), the statistical model is

$$\begin{aligned} g[E(y_{ijk})] = b_0&+ b_{1i} + b_{2ik} + b_{3} Exposure_{ijk} + b_{4} Condition_{ijk} + b_{5} TimeofDay_{ijk} + b_6 SecondDay_{ijk}\\&+ b_7 SaO2_{ik} + b_8 BetaBlocker_i + b_9 Pacemaker_i + b_{10} Exposure_{ijk} \times Condition_{ijk}, \end{aligned}$$

where $E(y_{ijk})$ is the mean of the binary presence or count outcome, and *g*() is the logit or log link (logit for presence (0/1) of any ectopic beats, log for the count of ectopic beats). $Exposure_{ijk}$, $Condition_{ijk}$, and $SecondDay_{ijk}$ are binary variables indicating the measurement occurred on the exposure day ($Exposure_{ijk} = 1$ if randomized to flight condition, 0 if control), during the activation of the chamber ($Condition_{ijk} = 1$ if post-activation, 0 if pre-activation), and on the second day in the chamber ($SecondDay_{ijk} = 1$ if second day, 0 if first day). $BetaBlocker_i$ and $Pacemaker_i$ are binary variables indicating beta blocker use and the presence of a pacemaker, respectively, and $SaO_{2ik}$ is the baseline blood oxygen saturation. The participant-specific intercept, $b_{1i}$, accounts for person-level variability, while the participant-exposure specific intercept, $b_{2ik}$, adjusts for within exposure-day variability. For the presence and rates of ectopic beats, we fit the above model to data from all subjects, as well as to subgroups consisting separately of subjects within the cardiac group, non-smokers, smokers, participants ages 65 and older, and participants less than age 65, respectively. Ratios of ORs or RRs for ectopy during post- versus pre-condition comparing flight and control days were calculated as $\exp (b_{10})$ with associated 95% confidence intervals.

We examined the impact of time in the chamber on ectopy using Binomial mixed effects models for the presence of ectopic beats and Negative Binomial mixed effects models for the rate. $Duration_{ijk}$ denotes duration of the experimental condition. The model for the effect of time is

$$\begin{aligned} g[E(y_{ijk})] = b_0&+ b_{1i} + b_{2ik} + b_{3} Exposure_{ijk} + b_{4} Duration_{ijk} + b_{5} TimeofDay_{ijk} + b_6 SecondDay_{ijk}\\&+ b_7 SaO2_{ik} + b_8 BetaBlocker_i + b_9 Pacemaker_i + b_{10} Exposure_{ijk} \times Duration_{ijk}, \end{aligned}$$

We also controlled for time of day, being accustomed to the chamber, baseline SaO_2_, beta blocker use, and presence of a pacemaker. The link function, *g*(), is the same as previously defined. The time-varying change in OR ratio or RR ratio for ectopy comparing exposure to control conditions was calculated as $\exp (b_{10})$. Associations were estimated using the glmmTMB function of the glmmTMB package in R version 3.6.1^16,17^.

## Results

We report participant characteristics among the entire study population and among smokers, non-smokers, and the cardiac group (Table 1). Many participants were current or former smokers (78%), 61% were male, 86% had baseline oxygen readings over 95%, and the mean age of participants was 63 years (66 among non-smokers, 61 among smokers and the cardiac group).

**Table 1:** Chamber study participant characteristics with descriptive statistics as frequencies and percentages or means and quartiles.

	All (N = 36)	Noncardiac/nonsmoker (N = 14)	Cardiac (N = 8)	Smoker (N = 14)
Male	22 (61.1%)	7 (50.0%)	6 (75.0%)	9 (65.3%)
Age (years)	63.2 (57.2, 69.2)	66.4 (61.6, 70.9)	61.4 (55.4, 69.2)	61.0 (56.7, 65.6)
Age 65 and older	13 (36.1%)	6 (42.9%)	3 (37.5%)	4 (28.6%)
White	27 (75.0%)	13 (92.9%)	6 (75.0%)	8 (57.1%)
Ever smoker	28 (77.8%)	8 (57.1%)	6 (75.0%)	14 (100.0%)
Body mass index	28.1 (24.2, 31.1)	28.3 (25.7, 30.0)	29.8 (28.4, 33.3)	27.1 (24.1, 30.2)
Diabetes	6 (16.7%)	1 (7.1%)	5 (62.5%)	0 (0.0%)
Asthma	3 (8.3%)	1 (7.1%)	1 (12.5%)	1 (7.1%)
COPD	3 (8.3%)	1 (7.1%)	2 (25.0%)	0 (0.0%)
Baseline SaO$_2$ >95	31 (86.1%)	9 (64.3%)	8 (100.0%)	14 (100.0%)
Beta-blocker use	13 (36.1%)	3 (21.4%)	7 (87.5%)	3 (21.4%)
Pacemaker use	8 (22.2%)	0 (0.0%)	8 (100.0%)	0 (0.0%)

SaO$_2$ denotes oxygen saturation.

We also present the counts of participants experiencing at least one VE or SVE according to study condition in Table 2. More participants experienced VE and SVE couplets than runs, regardless of treatment. The number of participants experiencing VE or SVE between pre- and post-condition are not noticeably different. This table, however, only presents the raw number of patients experiencing ectopy and not the presence or rate over the duration of the flight.

**Table 2:** Number of participants who experienced ectopic beats during pre- and post-condition, according to flight or control conditions.

	Control		Flight	
	Pre-condition	Post-condition	Pre-condition	Post-condition
VE couplets	22	22	22	22
VE runs	2	5	4	6
SVE couplets	29	27	29	30
SVE runs	8	9	4	9

Measurements were recorded before the start of the flight or control condition (pre-condition) and after conditions commenced (post-condition).

*SVE* supraventricular, *VE* ventricular.

We found that the presence of any VE did not differ significantly at the 0.05 level between flight and control conditions in adjusted models. For example, we observed a OR Ratio of 1.21 (Table 3; 95% CI: 0.81, 1.82) for VE couplets. The occurrence of SVE was significantly higher, also at the 0.05 level, during flight than control conditions (Table 3; OR ratio = 1.77, 95% CI: 1.21, 2.59 for couplets, OR ratio = 3.69, 95% CI: 1.37, 9.93 for runs).

The rates of VE and SVE were higher during flight conditions (Table 3; RR ratio = 1.25, 95% CI: 1.03, 1.52 for VE couplets, RR ratio = 1.63, 95% CI: 1.08, 2.46 for VE runs, RR ratio =1.76, 95% CI: 1.39, 2.22 for SVE couplets, RR ratio = 3.19, 95% CI: 1.22, 8.29 for SVE runs). Results in Table 3 are based on all the available data. The presence and rate of ectopy did not differ significantly among the cardiac, smoker, and non-cardiac non-smoker groups, or when subjects were categorized according to age (65+, or younger than 65; data not shown due to small subgroup sizes).

**Table 3:** Adjusted change in the odds ratio (OR) for the presence model and the adjusted change in the rate ratio (RR) for the rate model.

	Presence model		Rate model	
	OR ratio (95% C.I.)	P value	RR ratio (95% C.I.)	P value
VE couplets	1.21 (0.81, 1.82)	0.3531	1.25 (1.03, 1.52)	0.0257
VE runs	1.25 (0.39, 4.01)	0.7037	1.63 (1.08, 2.46)	0.0190
SVE couplets	1.77 (1.21, 2.59)	0.0034	1.76 (1.39, 2.22)	<0.0001
SVE runs	3.69 (1.37, 9.93)	0.0096	3.19 (1.22, 8.29)	0.0176

Couplet models are adjusted for time of day, order of conditions, baseline SaO$_2$, beta blocker use, and presence of a pacemaker. Run models are adjusted for time of day, order of conditions, beta blocker use, and presence of a pacemaker due to lack of convergence for SaO$_2$.

*SVE* supraventricular, *VE* ventricular, *CI* confidence interval.

**Figure 1:**
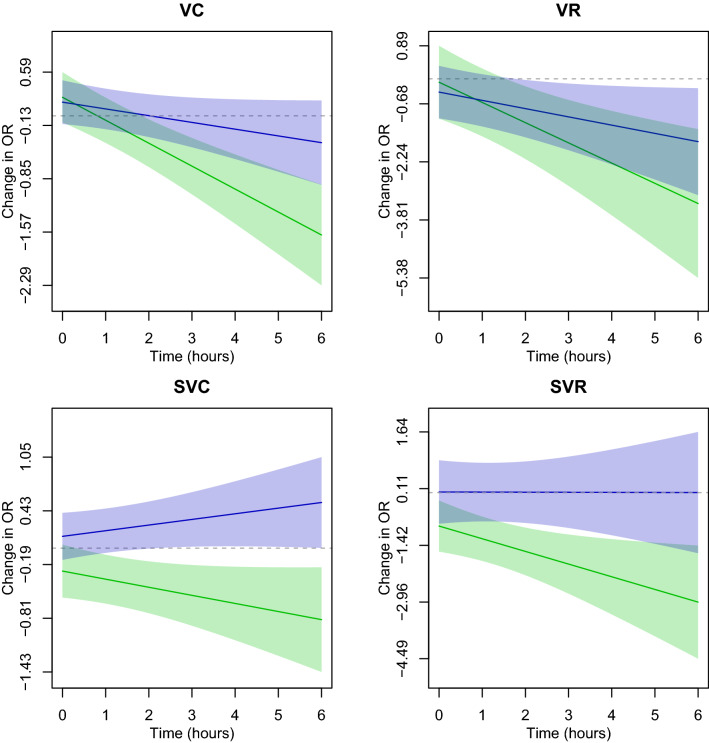
Time-varying change in the odds ratio (OR) between flight vs. control conditions and ectopy: ventricular couplets (VC), ventricular runs (VR), supraventricular couplets (SVC), and supraventricular runs (SVR). The OR comparing post- to pre-condition for simulated flight is in blue; the control day is in green. Solid lines depict the estimated OR while bands represent 95% confidence intervals. Estimates are from the duration model for the presence of ectopy.

**Figure 2:**
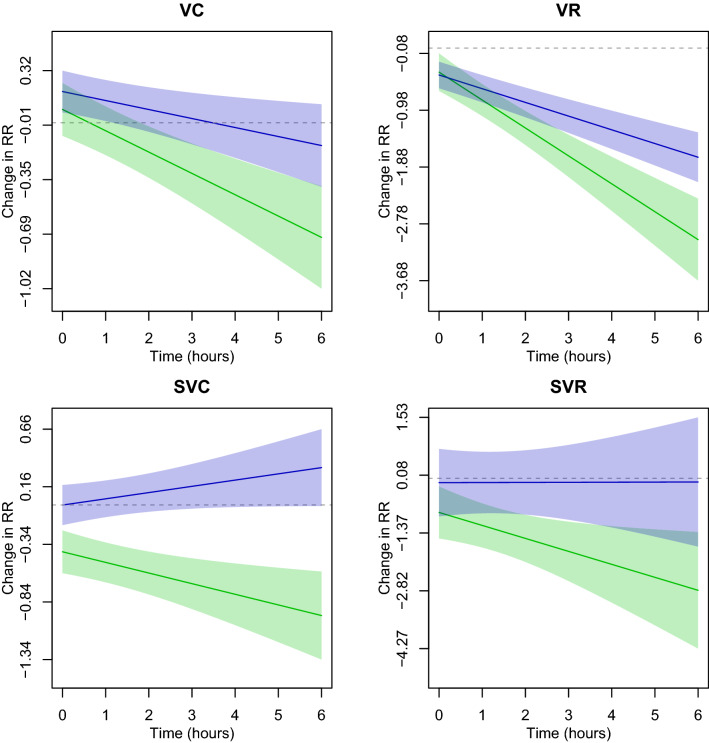
Time-varying change in rate ratio (RR) between flight vs. control conditions and ectopy: ventricular couplets (VC), ventricular runs (VR), supraventricular couplets (SVC), and supraventricular runs (SVR). The RR comparing post- to pre-condition for simulated flight is in blue; the control day is in green. Solid lines depict the estimated odds ratio while bands represent 95% confidence intervals. Estimates are from the duration model for the rate of ectopy.

Time-varying associations for the presence and rate of ectopy are presented in Figs. 1 and  2. We observe a pattern of an increasing OR ratio and RR ratio—that is to say presence and rate—of ectopy comparing flight to control conditions. These trends rise to statistical significance at the 0.05 level starting after about an hour in the chamber for SVE couplets when examining presence of ectopic beats. When examining the rate of ectopic beats, statistically significant differences between flight and control conditions began immediately for SVE couplets, at approximately an hour into flight for SVE runs, and after approximately two hours into a flight for VE runs, increasing throughout the duration of the flight condition.

## Discussion

In our chamber study of hypoxic flight conditions among passengers over age 50, we observed a higher rate of ventricular ectopy and a higher presence and rate of supraventricular ectopy during simulated flight conditions. These associations increased over the duration of the flight. Previous studies, including within our study population, report associations between flight and heart rate or heart rate variability^8–10^. However, to our knowledge, this is the first study to describe VE and SVE during controlled flight conditions. These results suggest that air travel may present cardiovascular stress to older and susceptible passengers and may have important implications for informing public health and medical guidelines. Although aircraft are typically pressurized to 8000 feet altitude, we observed higher rates of VE and SVE at 7000 feet altitude.

Prognoses for patients experiencing VE and SVE are, generally, mixed and there is uncertainty regarding the identification of patients who are at highest risk^6,7,18^. Ectopic beats are common and associated with a range of factors including age, height, blood pressure, heart disease, and smoking^18^. Patients experiencing VE or SVE are frequently asymptomatic^7,18^, however the presence of VE can, in some cases, indicate a susceptibility toward the development of arrhythmias^7^. Further, excessive SVE is associated with increased risk of developing atrial fibrillation and poor prognoses^6^. Increased frequency of VE or SVE may also be a risk factor for heart failure and death^18^. In our study, though we could not formally adjust for every person-level variable that may differ between flight and control conditions and affect VE or SVE occurrence or rate, our randomized crossover study design uses the participants as their own controls both within day (pre-condition vs post-condition) and between days (exposure vs control). Such a design, coupled with our statistical modeling, intrinsically controls for many person-level variables^19^. Furthermore, we formally adjusted for baseline oxygen saturation, beta blocker medications, and the presence of a pacemaker.

The changes in VE and SVE presence and rate we observed were time-varying and increased relative to control conditions for the entire duration of the flight condition, showing the importance of studying the effects of prolonged flight durations. According to established medical guidelines, most of the participants in our study presenting with VE or SVE would have been considered low risk for hypoxia given their baseline oxygen saturations^20^. People in the general population who are passengers on commercial aircraft (versus our study), however, may vary substantially in terms of their baseline oxygen saturation, respiratory and pulmonary physiology, and cardiac or respiratory symptoms.

Our participants were all over age 50, thus our results suggest that this group may show signs of cardiac stress at flight altitudes. Implications for younger passengers or crew are unclear based on our study, though we found that associations did not differ based on age being over 65, cardiac condition, or smoking status. Studies have also reported associations between flight, heart rate, and heart rate variability among young passengers^8^ and crew^9^, albeit minimally in the latter. It is plausible that those over age 50 may react differently to flight, however, because of physiological and immunological alterations in cardiac or respiratory system physiology and immunology^21^. The average age for flight attendants in the U.S. is 48.3 years^22^, and likely to increase due to COVID-related furloughs^23^. The retirement age for pilots also increased from 60 to 65 years, as of 2007^24^. While our study only examines the health effects on passengers, studying crew is important given their exposure profile and frequency, increased psychological and metabolic demands during flight compared to passengers, and the chronic stress, circadian disruption, fatigue, and sleep deprivation experienced by crew^25^.

Both a strength and limitation of our study is our focus on oxygen levels as separate from other flight exposures. An Airliner Cabin Environment Research report demonstrated substantial oxygen desaturation among these participants during flight conditions, with approximately half of participants exhibiting moderate hypoxia (desaturation to 90% or below), and most participants experiencing mild to moderate hypoxia (desaturation to 95% or below)^13^. Focusing on a specific exposure is a strength that helps elucidate our understanding of a driver of physiologic effects in flight. Altitude-induced hypoxia is known to acutely increase heart rate, stroke volume, and coronary blood flow but it does not appear to cause arrhythmias unless the ascent results in acute mountain sickness^26^. Heart rate, stroke volume, and coronary blood flow do gradually decrease to pre-ascent levels over a period of 2 to 10 days^26^. However it is important to note that these observations were made on ascents to high altitude that were more gradual than the ascents made during commercial flight. Moreover, flights are shorter periods of time than the 2- to 10-day period during which these metrics decrease. Learning more about physiological responses to flight conditions is necessary given the confluence of many stressors during flight. These stressors include circadian disruption which may cause arrhythmias^27^, noise, chemicals such as jet fuel and engine exhaust, and the psychological stress of flying which may causes arrhythmias as well^28^.

The Aerospace Medical Association recommends research into the effects of mild hypoxia for passengers and crew^29^. Larger chamber studies with more statistical power, real-time study of flight through wearable sensors and apps, and comprehensively examining cardiac and respiratory indices are necessary. The effects of flight exposures on acute cardiovascular events have not been well studied, yet passengers are being exposed to flights of increasing duration^30^. Recent evidence also suggests a possible increased risk in cardiac disorders amongst those previously infected with SARS-CoV-2^31^.

## Conclusion

In light of the Aerospace Medical Association recommendations, increasingly at-risk passengers, and our study’s findings, more research is necessary to better understand associations between flight, arrhythmias, and cardiovascular risk and to inform medical, public health, and airplane design guidelines regarding protections for passengers and crew. Our findings will guide research regarding cardiovascular effects of flight that elucidate relationships between flight, arrhythmias, cardiac events, and hypoxic pathways. Finally, our study utilized a cabin altitude of 7000 feet. While newer aircraft are capable of pressurizing the cabin to altitudes at or below this mark, the vast majority of commercial aircraft in service cannot. Future studies, conducted at lower altitudes, are therefore warranted to identify a safer margin for better protecting the health of susceptible passengers.

## Data Availability

The dataset used during the current study is available from the corresponding author upon reasonable request.
